# Post-treatment stability after insertion of CAD/CAM fabricated or Conventional fixed orthodontic retainers: a two-year follow-up

**DOI:** 10.1007/s00784-025-06368-4

**Published:** 2025-05-10

**Authors:** Maike Tabellion, Julia Simgen, Jörg Alexander Lisson

**Affiliations:** Department of Orthodontics (G56), Saarland University, Kirrberger Strasse 100, 66424 Homburg, Saar, Germany

**Keywords:** CAD/CAM, Fixed retainers, Stability, Intercanine distance, Little´s irregularity index

## Abstract

**Objective:**

Since relapse after orthodontic treatment and stability and failure of CAD/CAM fabricated and Conventional fixed retainers are widely discussed, this study investigated and compared two-year post-treatment stability after insertion of a CAD/CAM fabricated or Conventional fixed retainer in the lower jaw.

**Materials and methods:**

Digitized dental casts or intraoral scans of *n* = 60 patients were used for data acquisition. The patients were divided into two groups according to the retention protocol: CAD/CAM fabricated fixed retainer (*n* = 30, mean age 16.97 ± 5.74 years) and Conventional fixed retainer (*n* = 30, mean age 15.70 ± 4.19 years). The evaluation included established procedures for dental measurements of the mandible (Intercanine Distance and Little´s Irregularity Index) before orthodontic treatment, at the end of orthodontic treatment, when the fixed retainer was inserted and two years after the insertion of the fixed retainer. All retainers were inserted by the same practitioner. Complications were recorded. Statistics included Shapiro-Wilk-, T- and Friedman-Tests. The level of significance was set at *p* < 0.05.

**Results:**

In patients with Conventional fixed retainers the change of the Intercanine Distance between insertion of the fixed retainer and two years afterwards was significantly more pronounced than in patients with CAD/CAM fabricated fixed retainers (ICD: Δ CAD/CAM_t1−t2_: -0.03 ± 0.22 mm; Δ Conventional_t1−t2_: 0.12 ± 0.29 mm). Stability of Intercanine Distance was less in patients with Conventional fixed retainers. The change of Little´s Irregularity Index was not significant between the groups. In two patients with a Conventional fixed retainer a bonding surface was renewed within the first three months.

**Conclusions:**

Within two years, CAD/CAM fabricated fixed retainers showed less relapse of Intercanine Distance and fewer complications than Conventional fixed retainers.

**Clinical relevance:**

Considering the amount of relapse and the differences in complications, CAD/CAM fabricated fixed retainers and Conventional fixed retainers are useful appliances for stabilization of treatment results with favorable stability. Fabrication of CAD/CAM based fixed retainers is aside from that timesaving compared to Conventional fixed retainers.

## Introduction

To prevent relapse after orthodontic tooth movement, the retention phase should be seen as an important part of treatment. Retention appliances and protocols are widely discussed [1, 10, 14, 23, 25, 31]. Fixed retainers are attached to the lingual surface of mainly canines and/or incisors. Removable retainers are acrylic plates or vacuum formed splints [16, 17]. Since the 1970s, fixed retainers have gained fame increasing in recent years and been widely discussed [9, 30]. There were major concerns concerning plaque accumulation, but recent studies stated, that fixed retainers are not causing dental, gingival or periodontal diseases, but poor oral hygiene does [4, 5, 19]. Nonetheless, relapse after orthodontic treatment is complex and multifactorial [32]. Failure rate of fixed retainers and the risk of relapse due to tension in periodontal fibers cannot be predicted and are described with 3–90% in the lower arch [4, 6, 17, 30]. The final occlusion and the compliance of the patient can also affect stability [19]. Besides that, tooth movement after orthodontic treatment is seen as a normal change due to aging and occurs in patients without previous orthodontic treatment as well. Retainers are therefrom used to prevent relapse after orthodontic treatment or unwanted tooth movement due to aging [17]. Among orthodontists, different opinions concerning the need of retention, the type of the retainer and the time of the retention phase exist [3]. Materials and methods concerning retainers are evolving continuously and new alternatives are arising. However, standardized retention protocols are missing [3]. Computer- aided design and manufacturing (CAD/CAM) technology has been used for fixed orthodontic retainers using different materials [7]. CAD/CAM fabricated fixed retainers have been discussed as feasible alternatives to conventional, manually bent fixed retainers [28]. There are some studies comparing CAD/CAM fabricated and conventional fixed retainers six to twelve months after insertion [12, 18, 20, 21]. Fixed retainers may fail many years after orthodontic treatment [28]. Therefrom, it is important to evaluate their survival in longer terms. To our knowledge, the literature lacks studies that evaluate stability of orthodontic treatment results and fixed retainers in longer terms. This study adds value to the current literature by means of evaluating different materials and methodologies for fixed retainers in the lower jaw for at least two years, inserted by one clinician.

### Aims of the study

The aims of this study were the evaluation and comparison of two-years post-treatment stability after the insertion of a CAD/CAM fabricated or Conventional fixed retainer in the lower jaw inserted by one clinician. The purpose was to evaluate dental changes. Feasible complications in conjunction with CAD/CAM fabricated or Conventional fixed retainers should be considered.

## Materials and methods

### Patients

Between July 2018 and December 2022, in *n* = 67 patients fixed retainers were inserted in the mandible by the same practitioner. *N* = 7 patients were excluded from the study, since they did not come back for a follow-up.

**Fig. 1:**
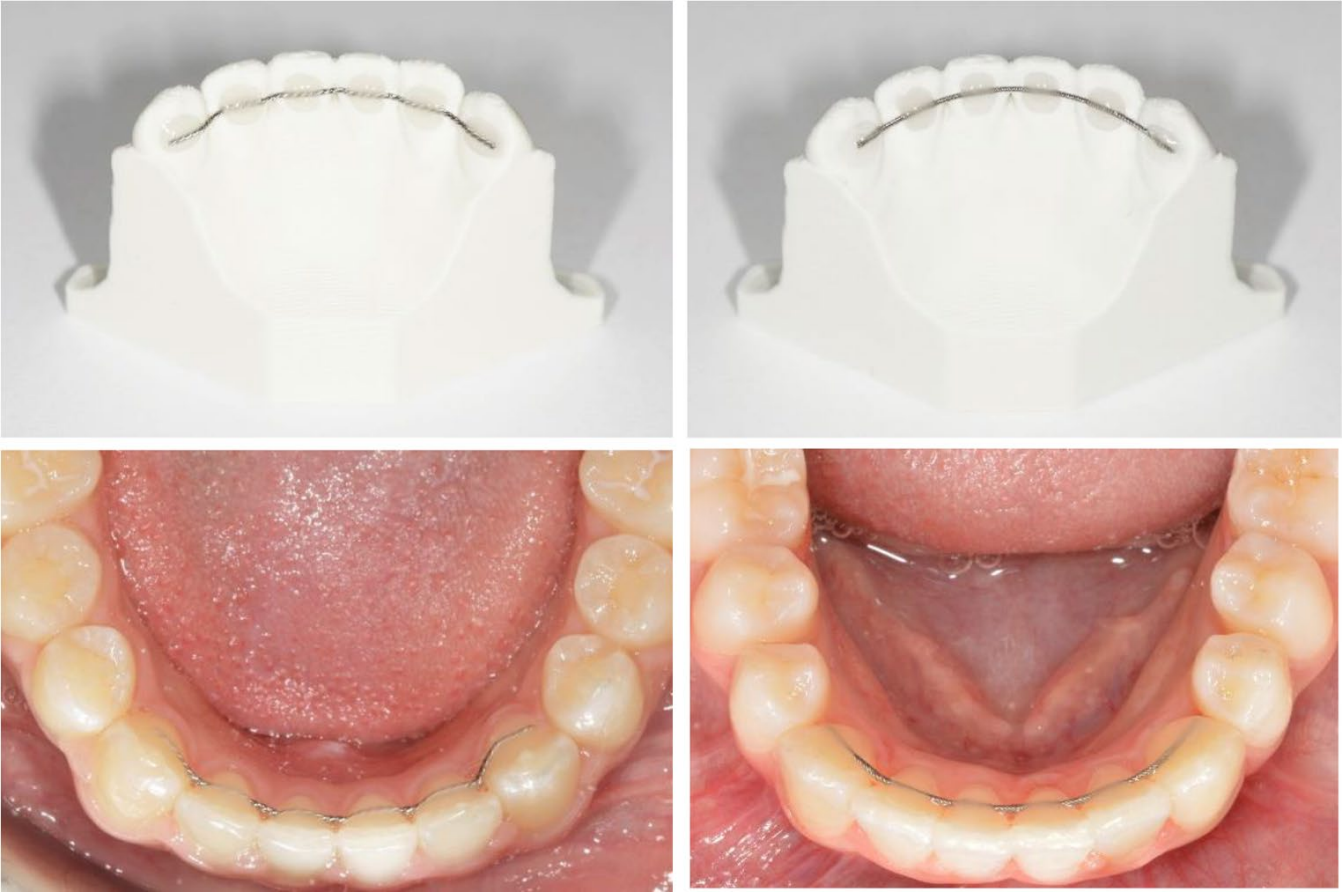
Overview of the mandibular fixed retainers on dental casts and in situ: CAD/CAM (left) and conventional (right)

The remaining *n* = 60 patients were divided into two groups (CAD/CAM fabricated fixed retainer or Conventional fixed retainer – Fig. 1), and compared to each other. The study was planned retrospectively. Digitized dental casts or intraoral scans by means of a TRIOS scanner (3Shape A/S, Copenhagen, Denmark) of the 60 non-syndromic patients (30 CAD/CAM fabricated fixed retainer, 30 Conventional fixed retainer) at the age of 16.97 ± 5.74 years (CAD/CAM fabricated fixed retainer) and 15.70 ± 4.19 years (Conventional fixed retainer) were identified and analyzed. Due to sample size, a gender division was not performed. All patients were diagnosed and treated exclusively at Saarland University Hospital. None of the patients needed extraction of premolars during treatment. The retainers were bonded on all six teeth from canine to canine in the mandible at the day of debonding. None of the patients received an additional, removable retention appliance.

### Inclusion/exclusion criteria

Inclusion criteria included completed orthodontic treatment with insertion of a fixed retainer at the end of treatment. All fixed retainers should have been inserted by the same practitioner. As a precondition, diagnostic data including dental casts or intraoral scans had to be present. Data were extracted from before the beginning (t_0_) and the end of orthodontic treatment (t_1_) and two years after insertion of a fixed retainer (t_2_). Exclusion criteria included comorbid syndromes and genetic disorders.

### Control group

All patients with CAD/CAM fabricated fixed retainers (*n* = 30) were matched with patients with Conventional fixed retainers (*n* = 30) regarding age and treatment protocol. Both groups received prior orthodontic treatment with brackets. Since many of our patients did not opt for fixed retainers due to financial reasons, Conventional fixed retainers were used in our clinic until 2020, and inclusion criteria was that the same practitioner inserted the fixed retainers, sample size determination was only partially possible. Against this background, we collected *n* = 30 patients with Conventional fixed retainers between 2018 and 2020. Since we changed to CAD/CAM fabricated fixed retainers in 2020, the sample size of this group matching the inclusion criteria until 2022 was *n* = 37 and *n* = 7 patients were excluded from the study. The mean treatment duration of patients receiving a CAD/CAM fabricated fixed retainer was 34.47 ± 1.28 months. The mean treatment duration of patients receiving a Conventional fixed retainer was 40.93 ± 19.10 months.

### CAD/CAM fabricated fixed retainer

**Fig. 2:**
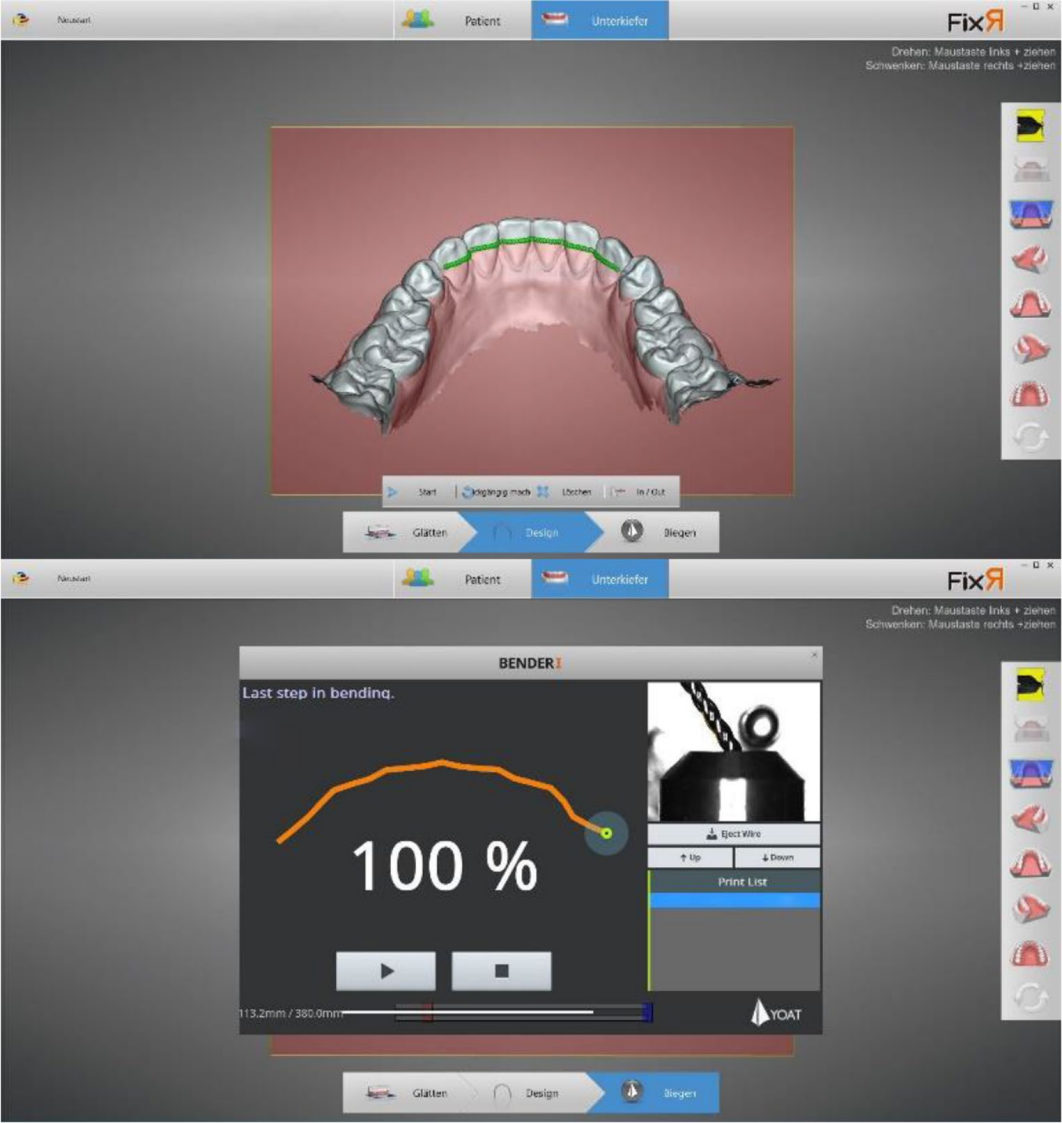
Overview of the proceeding of a custom-bent fixed retainer using the in-office CAD/CAM system with the software FixR and the BenderI machine

Fixed retainers were custom-bent using multistranded Dentaflex wires (dentaflex^®^, ⌀ 0.50 mm, Dentaurum GmbH & Co., Ispringen, Germany) the in-office CAD/CAM system with the software FixR and the BenderI machine (YOAT Corp., Lynwood, USA) (Fig. 2). All patients presented themselves for a quarterly recall for two years after insertion of the retainer.

### Conventional fixed retainer

Fixed retainers were bent manually by a dental technician by means of a dental cast of the lower jaw of the patient using preformed archwires (RESPOND^®^ 0.0170, Ormco Corp., California, USA). All patients presented themselves for a quarterly recall for two years after insertion of the retainer.

### Insertion technique of fixed retainers

CAD/CAM fabricated fixed retainers or Conventional fixed retainers were bonded on the lingual surfaces of the anterior teeth of the mandible. The proceeding was: cleaning of the teeth, application of 35% phosphoric acid (Reliance Orthodontic Products, Itasca, USA) and application of bonding agent (Transbond™ MIP, 3 M Germany GmbH, Neuss, Germany). The fixed retainers were placed secured with floss followed by application of light cure adhesive (Transbond LR, 3 M Germany GmbH, Neuss, Germany).

### Dental casts/intraoral scans measurement

A total of 180 mandibular dental casts or intraoral scans were available. Since 2020, we are using a TRIOS scanner (TRIOS^®^ 3, 3Shape A/S, Copenhagen, Denmark) for intraoral scans. Before 2020, the dental casts were digitized using a 3D-scanner orthoXScan (orthoX^®^ - DENTAURUM GmbH & Co. KG Ispringen, Germany). For linear measurements (Intercanine Distance and Little´s Irregularity Index) the software OnyxCeph^®^ 3TM (Image Instruments GmbH, Chemnitz, Germany) was used.

### Measuring technique

#### **Intercanine Distance and Little’s Irregularity Index** [24] (Fig. 3)

**Fig. 3:**
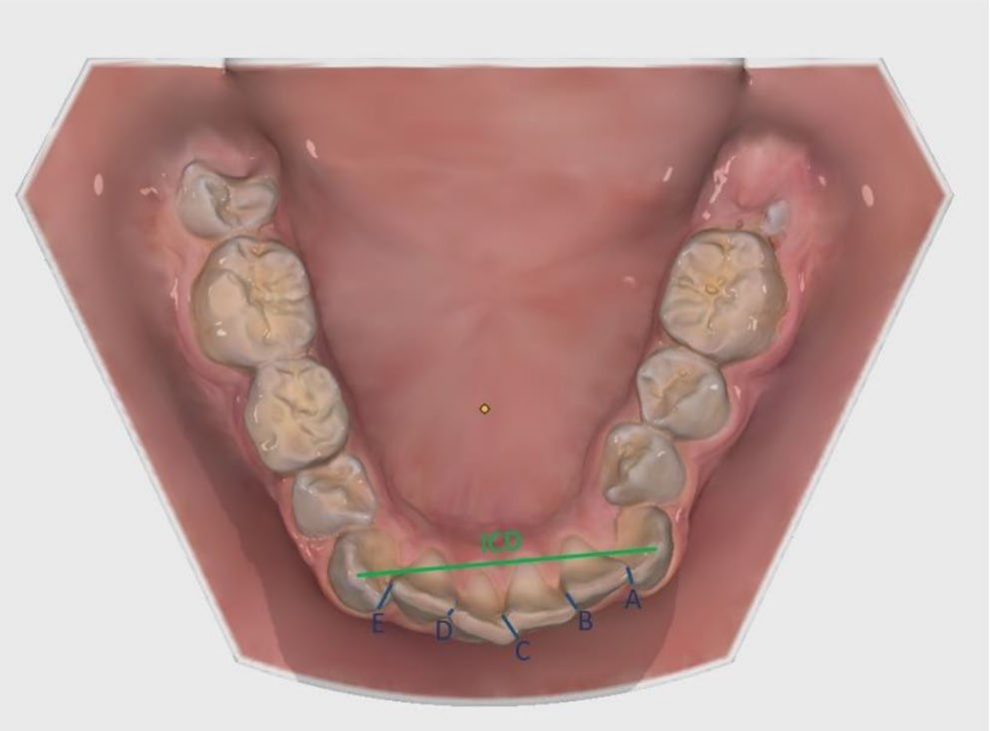
Overview of the linear measurements in the mandible for ICD (green) and LII (blue)

The distance between the cuspids of the canines (Intercanine Distance, ICD) of the mandible was measured. Linear distances from anatomic contact points to adjacent contact points of both canines and the four incisors of the mandible were measured (Distance A, B, C, D, E). The sum of the five measured distances represents Little´s Irregularity Index (LII) [24].

### Statistical analysis

Statistical analysis was performed with the SPSS software version 28 (IBM, Armonk, NY, USA). Statistics included Shapiro-Wilk-, T- and Friedman-Tests. The level of significance was set at *p* < 0.05. The significance level was defined as follows: *p* ≥ 0.05 not significant, *p* < 0.05 significant, *p* < 0.01 highly significant and *p* < 0.001 most highly significant. The effect sizes were tested using Cohen´s criteria (for r): 0.1–0.3 = small effect size and low correlation, 0.3–0.5 = moderate effect size and correlation, > 0.5 = large effect size and high correlation or Cohen´s criteria (for d): 0.2 = small effect size and low correlation, 0.5 = moderate effect size and correlation, 0.8 = large effect size and high correlation. For testing the interrater reliability the evaluation process was repeated by a second examiner two months after the first investigation to evaluate the impact of landmarking errors, which involved removing and replacing the markings. The differences were statistically analyzed using Pearson correlation test. The correlation *r* in the present study was > 0.7 for all measurements. Intrarater reliability was not tested, since a single examiner conducted the investigation and the degree of subjectivity existing despite time-shift should be minimized. According to power analysis using the software G*Power version 3.1.9 (HHU, Düsseldorf, Germany), with an effect size of 0.50 and an alpha level of 0.05 the actual power was 60.6%. An effect size of 0.5 was selected to verify moderate or large differences.

## Results

### Dental measurements

#### **CAD/CAM fabricated fixed retainers** (Table 1)

**Table 1:** CAD/CAM fabricated fixed retainers (*n* = 30) – measurements: distances [mm]. t_0_: pretreatment visit; t_1_: posttreatment visit, insertion of the fixed retainer, t_2_: two years after t_1_; *M* mean, *SD* standard deviation, ^a^T-test within group between t_0_-t_2_

T	t_0_	t_1_	t_2_	*P* value^a^ t_0_-t_1_	*P* value^a^ t_0_-t_2_	*P* value^a^ t_1_-t_2_
	M ± SD	M ± SD	M ± SD			
ICD	26.46 ± 1.49	26.72 ± 1.53	26.69 ± 1.57	0.246	0.289	0.458
LII	6.82 ± 2.93	1.89 ± 0.79	1.93 ± 0.75	<0.001	<0.001	0.526

In this group the Intercanine Distance increased between t_0_ and t_1_ and t_0_ and t_2_ (ICD: t_0_: 26.46 ± 1.49 mm; t_1_: 26.72 ± 1.53 mm; t_2_: 26.69 ± 1.57 mm; p_t0−t1_: 0.246; p_t0−t2_: 0.289) and decreased between t_1_ and t_2_ (ICD: t_1_: 26.72 ± 1.53 mm; t_2_: 26.69 ± 1.57 mm; p_t1−t2_: 0.458).

Little´s Irregularity Index decreased between t_0_ and t_1_ and t_0_ and t_2_ (LII: t_0_: 6.82 ± 2.93 mm; t_1_: 1.89 ± 0.79 mm; t_2_: 1.93 ± 0.75 mm; p_t0−t1_: <0.001, d_t0−t1_: 1.603; p_t0−t2_: <0.001, d_t0−t2_: 1.607) and increased between t_1_ and t_2_ (LII: t_1_: 1.89 ± 0.79 mm; t_2_: 1.93 ± 0.75 mm; p_t1−t2_: 0.526).

#### Conventional fixed retainers (Table 2)

**Table 2:** Conventional fixed retainers (*n* = 30) – measurements: distances [mm]. t_0_: pretreatment visit; t_1_: posttreatment visit, insertion of the fixed retainer, t_2_: two years after t_1_; *M* mean, *SD* standard deviation, ^a^T- or Friedman-test within group between t_0_-t_2_

T	t_0_	t_1_	t_2_	*P* value^a^ t_0_-t_1_	*P* value^a^ t_0_-t_2_	*P* value^a^ t_1_-t_2_
	M ± SD	M ± SD	M ± SD			
ICD	26.42 ± 2.32	26.57 ± 1.47	26.69 ± 1.65	0.710	0.496	0.026
LII	7.25 ± 2.41	2.23 ± 1.71	2.27 ± 1.66	<0.001	<0.001	0.519

In this group the Intercanine Distance increased between t_0_ and t_1,_ t_0_ and t_2_ and t_1_ and t_2_ (ICD: t_0_: 26.42 ± 2.32 mm; t_1_: 26.57 ± 1.47 mm; t_2_: 26.69 ± 1.65 mm; p_t0−t1_: 0.710; p_t0−t2_: 0.496; p_t1−t2_: 0.026, d_t1−t2_:0.428).

Little´s Irregularity Index decreased between t_0_ and t_1_ and t_0_ and t_2_ (LII: t_0_: 7.25 ± 2.41 mm; t_1_: 2.23 ± 1.71 mm; t_2_: 2.27 ± 1.66 mm; p_t0−t1_: <0.001, r_t0−t1_: 0.289; p_t0−t2_: <0.001, r_t0−t2_: 0.259) and increased between t_1_ and t_2_ (LII: t_1_: 2.23 ± 1.71 mm; t_2_: 2.27 ± 1.66 mm; p_t1−t2_: 0.519).

#### CAD/CAM fabricated versus Conventional fixed retainers – main differences (Table 3) and comparison at t_1_ (Table 4)

**Table 3:** CAD/CAM fabricated fixed retainers versus Conventional fixed retainers – measurements Δ: distances [mm]. t_0_: pretreatment visit; t_1_: posttreatment visit, insertion of the fixed retainer, t_2_: two years after t_1_; *M* mean, *SD* standard deviation, ^a^T-test between the groups between t_0_-t_2_

*Δ CAD/CAM fabricated fixed retainers*						
T	t_0_-t_1_	t_0_-t_2_	t_1_-t_2_			
	M ± SD	M ± SD	M ± SD			
ICD	0.26 ± 1.22	0.23 ± 1.18	-0.03 ± 0.22			
LII	-4.94 ± 3.08	-4.90 ± 3.05	0.04 ± 0.34			
Δ *Conventional fixed retainers*						
T	t_0_-t_1_	t_0_-t_2_	t_1_-t_2_	*P* value^a^ t_0_-t_1_	*P* value^a^ t_0_-t_2_	*P* value^a^ t_1_-t_2_
	M ± SD	M ± SD	M ± SD			
ICD	0.15 ± 2.18	0.27 ± 2.17	0.12 ± 0.29	0.805	0.930	0.024
LII	-5.02 ± 2.71	-4.98 ± 2.66	0.04 ± 0.22	0.912	0.911	1.000

**Table 4:** CAD/CAM fabricated fixed retainers versus Conventional fixed retainers – measurements: distances [mm]. t_1_: posttreatment visit, insertion of the fixed retainer; *M* mean, *SD* standard deviation, ^a^T-test between the groups at t_1_

*ICD*			
T	CAD/CAM t_1_	Conventional t_1_	P value^a^ t_1_
	M ± SD	M ± SD	
	26.72 ± 1.53	26.57 ± 1.47	0.724
*LII*			
T	CAD/CAM t_1_	Conventional t_1_	*P* value^a^ t_1_
	M ± SD	M ± SD	
	1.89 ± 0.79	2.23 ± 1.71	0.325

In patients with Conventional fixed retainers the change of Intercanine Distance between t_1_ and t_2_ was significantly more pronounced than in patients with CAD/CAM fabricated fixed retainers (ICD: Δ CAD/CAM_t1−t2_: -0.03 ± 0.22 mm; Δ Conventional_t1−t2_: 0.12 ± 0.29 mm; p_t1−t2_: 0.024, d_t1−t2_: 0.064). Stability of Intercanine Distance was less in patients with Conventional fixed retainers. The change of Little´s Irregularity Index was not significant between the groups. At t_1_, the Intercanine Distance and the Little´s Irregularity Index were almost indistinguishable between the groups.

### Fixed retainers complications

In one patient with a CAD/CAM fabricated fixed retainer, a change of the left mandibular canine was visible at t_2_. The retainer was preventively removed and renewed. In two patients with Conventional fixed retainers each of them had loosening of one bonding surface within three months after insertion of the retainer. The bonding surfaces could be renewed and were stable afterwards. Gingival recession or dental calculus were not present in any of the patients at t_2_.

## Discussion

Since the same practitioner should have inserted all fixed retainers of our study, the smaller number of 60 patients for this study was acceptable, especially regarding post-treatment stability evaluation after two years. Shortening of the two-year interval did not make sense, because stability should be assessed in longer terms. To our knowledge, this is the only study in existence covering a two-year follow up period concerning CAD/CAM fabricated fixed retainers compared to Conventional fixed retainers inserted by one clinician. Still, after division into different groups, necessary for comparison of different materials and methodologies of fixed retainers, the number of patients per group remained low. The different material, methodologies and insertion techniques of fixed retainers of different clinicians in existence did not allow the inclusion of casts or intraoral scans from other centers to increase numbers. However, especially CAD/CAM fabricated fixed retainers appear to be useful for stabilization of treatment outcomes. Alrawas et al. [2] compared 60 patients in their study for six months during retention phase with CAD/CAM NiTi, multi-stranded stainless steel, single stranded nickel-free titanium or vacuum-formed removable retainers in the lower jaw. The patients were approximately 20 years old at the beginning of the retention phase. They concluded that in all groups relapse was seen in anterior teeth, but regarding clinical failure rate, no differences were found between the groups. The results are comparable to our results in short-term. Furthermore, they observed less plaque accumulation and gingival inflammation in the CAD/CAM NiTi retainer group. Egli et al. [11] compared 64 patients with indirect or direct bonded fixed retainers in the lower jaw and described a failure rate of 40% within two years with 43% failure rate in the indirect bonding group and 37% in the direct bonding group occurring mainly during the first year. In our study, the failure rate was 3% in patients with CAD/CAM fabricated fixed orthodontic retainers and 6% in the patients with Conventional fixed retainers, but comparison of failure rates is difficult due to heterogeneity in bonding methods, dimensions and materials and differences in follow-up duration. Pulisaar et al. [27] evaluated 181 patients, 90 with CAD/CAM fabricated fixed retainers (Memotain^®^) and 91 with conventional multistranded fixed retainers of two centres 12 and 24 months after insertion of the retainers. They included maxillary retainers as well. Within 24 months, they described a failure rate of CAD/CAM fabricated fixed retainers with 42% and of conventional multistranded fixed retainers with 40%. In groups of failure-free patients, differences in Little´s Irregularity Index and arch dimensions were not remarked.

Schmid-Herrmann et al. [29] analyzed the accuracy of 41 retainers including CAD/CAM and hand-bent retainers by means of superimposition. They described CAD/CAM retainers with a good positioning accuracy in the mandible and good retention properties regarding posttreatment changes.

Tran et al. [32] compared a total of 43 patients divided into three different groups: CAD/CAM group with multistranded stainless steel retainers, lab group with the same multistranded stainless steel retainers and a chairside group with stainless steel Ortho-FlexTech wires in the lower jaw. After two years, they reported a significantly less reduction of intercanine width in patients with CAD/CAM multistranded stainless steel retainers compared to the lab and chairside group and a lower failure rate (21.4%). The sample size per group was smaller than in our study but results regarding stability of intercanine distance was similar. They used Dentaflex wires as well in the CAD/CAM and in the lab group and described the Dentaflex wires as more rigid compared to the Ortho-FlexTech wires and as more stabilizing concerning intercanine witdth. In our study, the CAD/CAM fabricated fixed retainer was less flexible as the Conventional fixed retainer as well. Muhareb et al. [26] stated in their review that concerning intercanine distance, arch length and failure rate, differences between CAD/CAM and conventional retainers were not significant regardless of follow up time or retainer wire type. Plaque index was reduced with CAD/CAM retainers after six months. Regarding intercanine distance and failure rate, the results of our study were similar.

Baysal et al. [8] reported, that rigid stainless steel wires are less easily deformed due to mastication forces or cleaning. An explanation concerning the lower failure rate of CAD/CAM fabricated fixed retainers using a bending machine can be that the bending machine is able to bend more precisely following the lingual contours leading to better interproximal adaption [32]. Hu et al. [13] concluded in their study, that the CAD/CAM method in fabricating lingual retainers is more stable and saves much time. They reported, that fabricating a lingual retainer in the lower jaw using the CAD/CAM method takes only half of the time compared to fabricating a conventional one. Concerning failure rate, insertion technique of the clinician can also play an important role, especially because bonding failure is the main reason why fixed retainers fail [15, 22, 32]. To minimize different insertion techniques, all fixed retainers of our study were inserted by one clinician. Moreover, patients’ compliance during mastication is important, since too much power can lead to failure of the fixed retainer as well [19].

### Limitations of the study

The number of patients of our study was acceptable. Nonetheless, a larger patient number could have been reached, but many patients could not defray the costs of fixed retainers or did not come back for a follow-up after insertion of the fixed retainer. Moreover, the retainers should be inserted by the same practitioner. Due to the smaller number of patients, a gender division was not performed during comparison of post-treatment stability. The recruitment duration was rather long, since only a few patients opt for fixed retainers. Some of our patients decided on removable retainers for financial reasons.

Clinical applicability of our study was limited due to the two-year follow-up, since retention needs to be seen as a long-term subject of decades.

Another limit of our study is the evaluation of Little´s Irregularity Index, since deviations of contact points were measured, but the Index does not consider inclination or vertical changes. At baseline, crowding was more in patients with Conventional fixed retainers, but the patients were randomly selected for the study.

Finally, post-treatment stability does not exclusively depend on final occlusion and duration of the retention phase, but also on the patient’s compliance concerning appropriate handling of a fixed retainer.

## Conclusion

Within two years, CAD/CAM fabricated fixed retainers showed less relapse of Intercanine Distance and fewer complications than Conventional fixed retainers. Nonetheless, considering clinical relevance, the actual amount of relapse and the difference in complications between CAD/CAM fabricated fixed retainers and Conventional fixed retainers are almost indistinguishable.

Fabrication of CAD/CAM based fixed retainers is aside from that timesaving compared to Conventional fixed retainers. Larger patient numbers and a longer follow-up examination, however, would have been favorable for a final assessment.

## Data Availability

No datasets were generated or analysed during the current study.

## References

[1] Al-Moghrabi D, Littlewood SJ, Fleming PS (2021) Orthodontic retention protocols: an evidence-based overview. Br Dent J 230:770–776. 10.1038/s41415-021-2954-7 34117437 10.1038/s41415-021-2954-7

[2] Alrawas MB, Kashoura Y, Tosun Ö, Öz U (2021) Comparing the effects of CAD/CAM nickel-titanium lingual retainers on teeth stability and periodontal health with conventional fixed and removable retainers: A randomized clinical trial. Orthod Craniofac Res 24:241–250. 10.1111/ocr.12425 32865325 10.1111/ocr.12425

[3] Andriekute A, Vasiliauskas A, Sidlauskas A (2017) A survey of protocols and trends in orthodontic retention. Prog Orthod 18(1):31. 10.1186/s40510-017-0185-x 28990138 10.1186/s40510-017-0185-x PMC5632597

[4] Arn ML, Dritsas K, Pandis N, Kloukos D (2020) The effects of fixed orthodontic retainers on periodontal health: A systematic review. Am J Orthod Dentofac Orthop 157:156–164e17. 10.1016/j.ajodo.2019.10.010 10.1016/j.ajodo.2019.10.010 32005466

[5] Artun J (1984) Caries and periodontal reactions associated with long-term use of different types of bonded lingual retainers. Am J Orthod 86:112–118. 10.1016/0002-9416(84)90302-6 10.1016/0002-9416(84)90302-6 6380296

[6] Aye ST, Liu S, Byrne E, El-Angbawi A (2023) The prevalence of the failure of fixed orthodontic bonded retainers: a systematic review and meta-analysis. Eur J Orthod 45:645–661. 10.1093/ejo/cjad047 37824794 10.1093/ejo/cjad047 PMC10687514

[7] Bardideh E, Ghorbani M, Shafaee H, Saeedi P, Younessian F (2023) A comparison of CAD/CAM-based fixed retainers versus conventional fixed retainers in orthodontic patients: a systematic review and network meta-analysis. Eur J Orthod 45:545–557. 10.1093/ejo/cjad033 37471113 10.1093/ejo/cjad033

[8] Baysal A, Uysal T, Gul N, Alan MB, Ramoglu SI (2012) Comparison of three different orthodontic wires for bonded lingual retainer fabrication. Korean J Orthod 42:39–46. 10.4041/kjod.2012.42.1.39 23112930 10.4041/kjod.2012.42.1.39 PMC3481967

[9] Bjering R, Vandevska-Radunovic V (2018) Occlusal changes during a 10-year posttreatment period and the effect of fixed retention on anterior tooth alignment. Am J Orthod Dentofac Orthop 154:487–494. 10.1016/j.ajodo.2017.12.015 10.1016/j.ajodo.2017.12.015 30268259

[10] Chawla R, Ansari FM, Chekka M, Akhilesh M, Deepak VA, Varma Datla PK, Ravuri P (2024) Assessment of orthodontic retention protocols and their effects on treatment stability. J Pharm Bioallied Sci 16:2403–2406. 10.4103/jpbs.jpbs_270_24 10.4103/jpbs.jpbs_270_24 PMC11426873 39346320

[11] Egli F, Bovali E, Kiliaridis S, Cornelis MA (2017) Indirect vs direct bonding of mandibular fixed retainers in orthodontic patients: comparison of retainer failures and posttreatment stability. A 2-year follow-up of a single-center randomized controlled trial. Am J Orthod Dentofac Orthop 151:15–27. 10.1016/j.ajodo.2016.09.009 10.1016/j.ajodo.2016.09.009 28024770

[12] Gelin E, Seidel L, Bruwier A, Albert A, Charavet C (2020) Innovative customized CAD/CAM nickel-titanium lingual retainer versus standard stainless-steel lingual retainer: A randomized controlled trial. Korean J Orthod 50:373–382. 10.4041/kjod.2020.50.6.373 33144526 10.4041/kjod.2020.50.6.373 PMC7642231

[13] Hu X, Ling J, Wu X (2019) The CAD/CAM method is more efficient and stable in fabricating of lingual retainer compared with the conventional method. Biomed J Sci Tech Res 18:1–4

[14] Hussain U, Kunwar SS, Khan UW, Alnazeh AA, Kamran MA, Alam S, Aziz A, Zaheen M, Pandis N, Campobasso A (2024) Can vacuum-formed retainers maintain arch dimensions and alignment compared to Hawley and fixed bonded retainers after treatment with fixed appliances? A systematic review and meta-analysis. Eur J Orthod 46:cjae040. 10.1093/ejo/cjae040 39177154 10.1093/ejo/cjae040

[15] Iliadi A, Kloukos D, Gkantidis N, Katsaros C, Pandis N (2015) Failure of fixed orthodontic retainers: A systematic review. J Dent 43:876–896. 10.1016/j.jdent.2015.05.002 25979824 10.1016/j.jdent.2015.05.002

[16] Jedliński M, Grocholewicz K, Mazur M, Janiszewska-Olszowska J (2021) What causes failure of fixed orthodontic retention? - systematic review and meta-analysis of clinical studies. Head Face Med 17:32. 10.1186/s13005-021-00281-3 34301280 10.1186/s13005-021-00281-3 PMC8306281

[17] Johnston CD, Littlewood SJ (2015) Retention in orthodontics. Br Dent J 218(3):119–122. 10.1038/sj.bdj.2015.47 25686428 10.1038/sj.bdj.2015.47

[18] Jowett AC, Littlewood SJ, Hodge TM, Dhaliwal HK, Wu J (2023) CAD/CAM nitinol bonded retainer versus a chairside rectangular-chain bonded retainer: A multicentre randomised controlled trial. J Orthod 50:55–68. 10.1177/14653125221118935 36062600 10.1177/14653125221118935 PMC10031634

[19] Kartal Y, Kaya B, Fixed Orthodontic Retainers A, Review (2019) Turk J Orthod 32:110–114. 10.5152/TurkJOrthod.2019.18080 31294414 10.5152/TurkJOrthod.2019.18080 PMC6605884

[20] Kartal Y, Kaya B, Polat-Özsoy Ö (2021) Comparative evaluation of periodontal effects and survival rates of memotain and five-stranded bonded retainers: A prospective short-term study. J Orofac Orthop 82:32–41. 10.1007/s00056-020-00243-5 32780168 10.1007/s00056-020-00243-5

[21] Knaup I, Wagner Y, Wego J, Fritz U, Jäger A, Wolf M (2019) Potential impact of lingual retainers on oral health: comparison between conventional twistflex retainers and CAD/CAM fabricated nitinol retainers: A clinical in vitro and in vivo investigation. J Orofac Orthop 80:88–96. 10.1007/s00056-019-00169-7 30778609 10.1007/s00056-019-00169-7

[22] Kučera J, Marek I (2016) Unexpected complications associated with mandibular fixed retainers: A retrospective study. Am J Orthod Dentofac Orthop 149:202-11. 10.1016/j.ajodo.2015.07.035 10.1016/j.ajodo.2015.07.035 26827976

[23] Kumar SO, Srinidhi S, Visshishta J, Sirisha S, Ranganathan S, Priya K, Moulvi S, Mani B (2024) Evaluation of orthodontic retention protocol among orthodontist and general Dentist-A Cross-Sectional survey. J Pharm Bioallied Sci 16:1588–1590. 10.4103/jpbs.jpbs_1099_23 10.4103/jpbs.jpbs_1099_23 PMC11174164 38882740

[24] Little RM (1975) The irregularity index: a quantitative score of mandibular anterior alignment. Am J Orthod 68:554–563. 10.1016/0002-9416(75)90086-x 1059332 10.1016/0002-9416(75)90086-x

[25] Meade MJ, Millett D (2013) Retention protocols and use of vacuum-formed retainers among specialist orthodontists. J Orthod 40:318–325. 10.1179/1465313313Y.0000000066 24297964 10.1179/1465313313Y.0000000066

[26] Muhareb LA, Talsania B, Al-Jewair T, CAD/CAM-based fixed lingual orthodontic retainers may be as effective as conventional fixed retainers (2024) J Evid Based Dent Pract 24(3):102008. 10.1016/j.jebdp.2024.102008 39174166 10.1016/j.jebdp.2024.102008

[27] Pullisaar H, Cattaneo PM, Gera A, Sankiewicz M, Bilińska M, Vandevska-Radunovic V, Cornelis MA (2024) Stability, survival, patient satisfaction, and cost-minimization of CAD/CAM versus conventional multistranded fixed retainers in orthodontic patients: a 2-year follow-up of a two-centre randomized controlled trial. Eur J Orthod 46(2):cjae006. 10.1093/ejo/cjae006 38394353 10.1093/ejo/cjae006 PMC10888518

[28] Roser CJ, Bauer C, Hodecker L, Zenthöfer A, Lux CJ, Rues S (2023) Comparison of six different CAD/CAM retainers vs. the stainless steel twistflex retainer: an in vitro investigation of survival rate and stability. J Orofac Orthop. 10.1007/s00056-023-00486-y 37378840 10.1007/s00056-023-00486-y PMC11861246

[29] Schmid-Herrmann CU, Schwieder T, Kahl-Nieke B, Koehne T (2025) Positioning accuracy and post-treatment changes in the mandibular arch after insertion of a CAD/CAM titanium retainer. Int J Comput Dent 28;0(0):0. 10.3290/j.ijcd.b5951470 10.3290/j.ijcd.b5951470 39871806

[30] Shim H, Foley P, Bankhead B, Kim KB (2022) Comparative assessment of relapse and failure between CAD/CAM stainless steel and standard stainless steel fixed retainers in orthodontic retention patients. Angle Orthod 92:87–94. 10.2319/121720-1015.1 34464438 10.2319/121720-1015.1 PMC8691474

[31] Steegmans PAJ, Cavagnetto D, Reynders RAM (2022) Which orthodontic retention protocol should I implement? A critical assessment of a randomised controlled trial. Evid Based Dent 23:162–165. 10.1038/s41432-022-0845-7 36526846 10.1038/s41432-022-0845-7

[32] Tran G, Rucker R, Foley P, Bankhead B, Adel SM, Kim KB (2024) Relapse and failure rates between CAD/CAM and conventional fixed retainers: a 2-year follow-up of a randomized controlled clinical trial. Eur J Orthod 46(1):cjad079. 10.1093/ejo/cjad079 38168815 10.1093/ejo/cjad079

